# Molecular Characterization and Biological Effects of a C-Type Lectin-Like Receptor in Large Yellow Croaker (*Larimichthys crocea*)

**DOI:** 10.3390/ijms161226175

**Published:** 2015-12-10

**Authors:** Jingqun Ao, Yang Ding, Yuanyuan Chen, Yinnan Mu, Xinhua Chen

**Affiliations:** 1 Key Laboratory of Marine Biogenetic Resources, Third Institute of Oceanography, State Oceanic Administration, Xiamen 361005, China; ajingqun@tio.org.cn (J.A.); dingyang@stu.xmu.edu.cn (Y.D.); chenyuanyuan@wolwobiotech.com (Y.C.); muyinnan@tio.org.cn (Y.M.); 2 Fujian Collaborative Innovation Center for Exploitation and Utilization of Marine Biological Resources, Key Laboratory of Marine Genetic Resources of Fujian Province, Xiamen 361005, China; 3 Laboratory for Marine Biology and Biotechnology, Qingdao National Laboratory for Marine Science and Technology, Qingdao 266071, China

**Keywords:** large yellow croaker *Larimichthys crocea*, C-type lectin-like receptor, hemagglutination, bacterial agglutination, antibacterial immunity

## Abstract

The C-type lectin-like receptors (CTLRs) play important roles in innate immunity as one type of pattern recognition receptors. Here, we cloned and characterized a C-type lectin-like receptor (LycCTLR) from large yellow croaker *Larimichthys crocea*. The full-length cDNA of LycCTLR is 880 nucleotides long, encoding a protein of 215 amino acids. The deduced LycCTLR contains a C-terminal C-type lectin-like domain (CTLD), an N-terminal cytoplasmic tail, and a transmembrane region. The CTLD of LycCTLR possesses six highly conserved cysteine residues (C1–C6), a conserved WI/MGL motif, and two sugar binding motifs, EPD (Glu-Pro-Asp) and WYD (Trp-Tyr-Asp). Ca^2+^ binding site 1 and 2 were also found in the CTLD. The LycCTLR gene consists of five exons and four introns, showing the same genomic organization as tilapia (*Oreochromis niloticus*) and guppy (*Poecilia retitculata*) CTLRs. LycCTLR was constitutively expressed in various tissues tested, and its transcripts significantly increased in the head kidney and spleen after stimulation with inactivated trivalent bacterial vaccine. Recombinant LycCTLR (rLycCTLR) protein produced in *Escherichia coli* BL21 exhibited not only the hemagglutinating activity and a preference for galactose, but also the agglutinating activity against two food-borne pathogenic bacteria *E. coli* and *Bacillus cereus* in a Ca^2+^-dependent manner. These results indicate that LycCTLR is a potential galactose-binding C-type lectin that may play a role in the antibacterial immunity in fish.

## 1. Introduction

Innate immune system plays a major role in the defense against invading microbial pathogens in bony fish [1]. Pathogen recognition is a crucial step for the initiation of immune responses [2,3]. The C-type lectin-like receptors (CTLRs) are a large family of pattern recognition receptors that bind to carbohydrates in a calcium (Ca^2+^)-dependent manner. These molecules generally contain at least one C-type lectin-like domain (CTLD), which forms a characteristic double-loop structure, disulfide-bond positions, and Ca^2+^-binding sites. However, many structurally homologous domains to the CTLD were shown not to be restricted to carbohydrate binding [3,4]. CTLRs serve many different biological functions in mammals, including pathogen-associated molecular pattern (PAMP) recognition, agglutination of microbial cells, phagocytosis, migration and apoptosis, and induction of inflammatory response [4,5,6,7,8].

At present, many fish CTLR genes have been identified, such as natural killer cell CTLR genes in cichlid fish (*Paralabidochromis chilotes*) and Nile tilapia (*Oreochromis niloticus*) [9], three kinds of CTLR (CTLR A, B, and C) genes in Atlantic salmon (*Salmo salar*) [10], lectin-like receptor genes in zebrafish (*Danio rerio*) [11,12], mannose receptor (MR) genes in blunt snout bream (*Megalobrama amblycephala*) and grass carp (*Ctenopharyngodon idella*) [13,14], and three CTLR-like genes in ayu (*Plecoglossus altivelis*) [15,16,17]. In ayu, a CTLR-like protein PaCD209L could bind Gram-negative and Gram-positive bacteria in the absence of Ca^2^^+^ and plays a role in the regulation of the phagocytosis and bacterial killing of monocytes/macrophages (MO/Mϕ) [16], while another CTLR (PaCTLRC) agglutinates several Gram-negative and Gram-positive bacteria in a Ca^2+^-dependent manner [17]. Blunt snout bream MR has been found to mediate phagocytosis of bacteria in macrophages in a Ca^2+^-dependent manner [14]. Furthermore, zebrafish CD209 homologue (lectin-like receptor) has been verified to participate in the initiation and development of adaptive immunity [18]. However, the characters and functions of most fish CTLRs remain unknown.

In this study, we first report the molecular characterization of a novel C-type lectin-like receptor (LycCTLR) from the large yellow croaker *Larimichthys crocea*. Tissue expression of LycCTLR under normal or bacterial vaccine-stimulated conditions was then analyzed. Furthermore, hemagglutinating activity, sugar binding specificity, and bacteria agglutinating activity of recombinant LycCTLR (rLycCTLR) protein were also investigated. Our results will be helpful for further understanding of characters and functional activity of C-type lectin-like receptors in fish.

## 2. Results

### 2.1. cDNA and Gene Structures of LycCTLR

**Figure 1:**
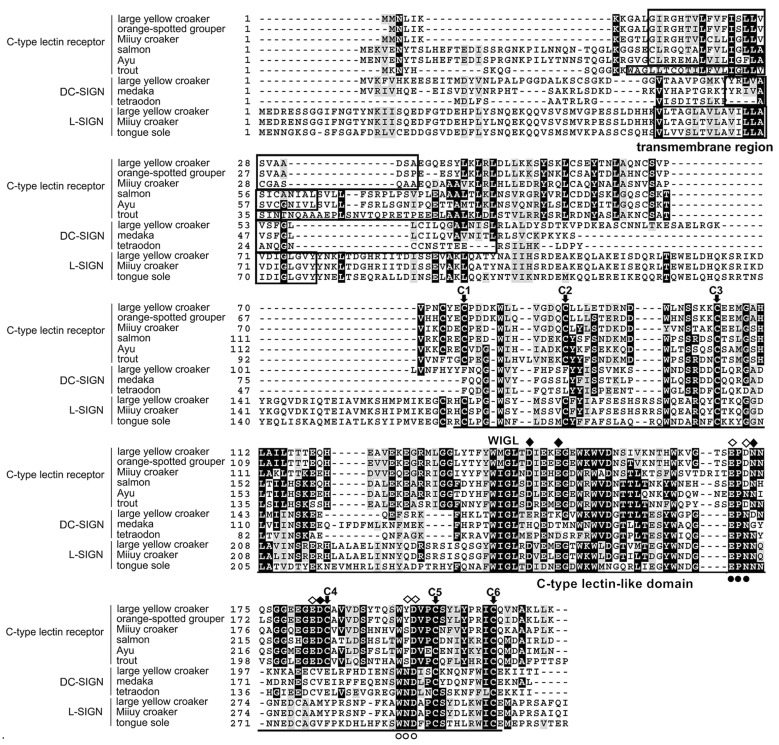
Alignment of deduced amino acid sequences of LycCTLR with other species CTLR, DC-SIGN, and L-SIGN molecules. Sequence alignments were obtained with ClustalX2 by the ClustalW method and conserved residues are shaded using BOXSHADE (v3.21, Swiss institute of bioinformatics, Swaziland). The transmembrane domain is boxed. The C-type lectin-like domain is underlined. The identical residues are indicated with black background, and similar residues are indicated with dark grey background. The six highly conserved cysteine residues are indicated by arrows. EPD motif and WYD motif are indicated with solid and hollow cycles below, respectively. Ca^2+^ binding site 1 are marked with solid diamond. Ca^2+^ binding site 2 are marked with hollow diamond.

The open reading frame of LycCTLR (GQ265786) is 648 nucleotides (nt), encoding a protein of 215 amino acids (aa), with a theoretical molecular weight of 24.3 kDa. The deduced LycCTLR contains a C-terminal C-type lectin-like domain (CTLD, residues 76–208), a short N-terminal cytoplasmic tail, and a transmembrane region (residues 12–34). Three potential glycosylation sites were found in the LycCTLR CTLD (N^65^CSV^68^, N^100^SSK^103^, and N^174^QSG^177^) (Figure S1). Multiple sequence alignment revealed that the CTLD of LycCTLR possesses six highly conserved cysteine residues (C1–C6), four of which (C3–C6 and C4–C5) are important for the formation of the internal disulphides, a conserved WI/MGL motif, and two sugar binding motifs, EPD (residues 170–172) and WYD (residues 194–196) (Figure 1). Moreover, Ca^2+^ binding site 1 (Asp^144^, Glu^14^^8^, Asn^174^, and Asp^183^) and Ca^2+^ binding site 2 (Glu^170^, Asp^172^, Glu^182^, Try^195^, and Asp^196^) were also identified in the CTLD (Figure 1).

**Figure 2:**
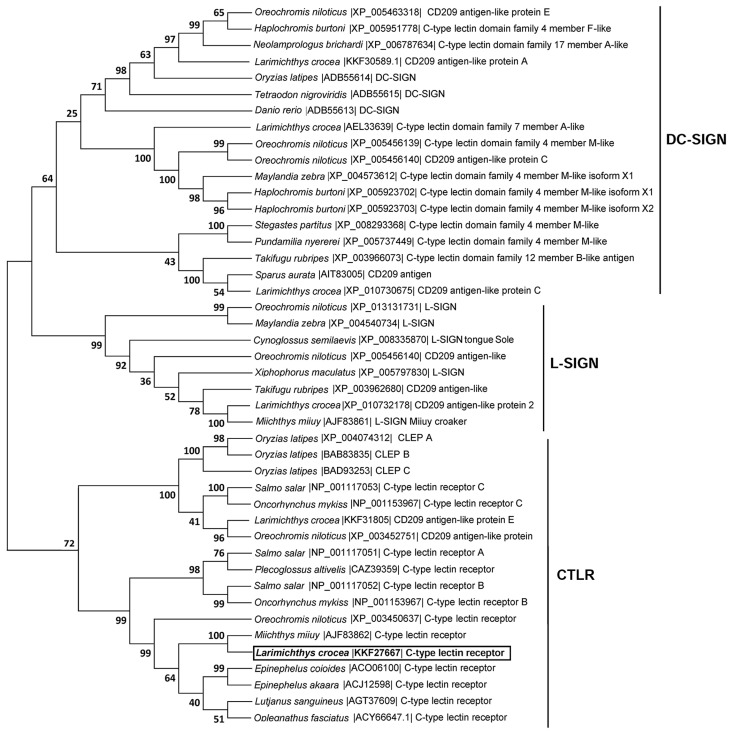
Phylogenetic tree based on the genetic distances of deduced amino acid sequences of fish CTLR, DC-SIGN, and L-SIGN. Deduced amino acid sequences of fish CTLR, DC-SIGN, and L-SIGN were aligned using CLUSTAL X, and the tree was constructed with the neighbour-joining method by MEGA 6.0 software [19]. Numbers on nodes represent frequencies with which the node is recovered per 100 bootstrap replications in a total of 10,000. The LycCTLR is boxed and in bold. The GenBank accession numbers of the sequences used here are shown in the figure.

Sequence alignments based upon amino acid identities showed that LycCTLR shares the highest sequence identity of 88.4% to miiuy croaker (*Miichthys miiuy*) CTLR, and a higher sequence identity to its homologues from tilapia, barred knifejaw (*Oplegnathus fasciatus*), humphead snapper (*Lutjanus sanguineus*), and orange-spotted grouper (*Epinephelus coioides*) (59.5%–62.3%). However, LycCTLR has a lower sequence identity of 14%–20% to fish C-type lectin domain-containing dendritic cell-specific ICAM-3-grabbing non-integrin (DC-SIGN) and Liver/lymph node-specific ICAM-3-grabbing non-integrin (L-SIGN) molecules (Table 1). Phylogenetic analysis shows that LycCTLR falls into a major clade formed by known fish CTLR molecules, separate from fish DC-SIGN and L-SIGN groups, suggesting that LycCTLR is a new member of fish CTLR family (Figure 2).

**Figure 3:**
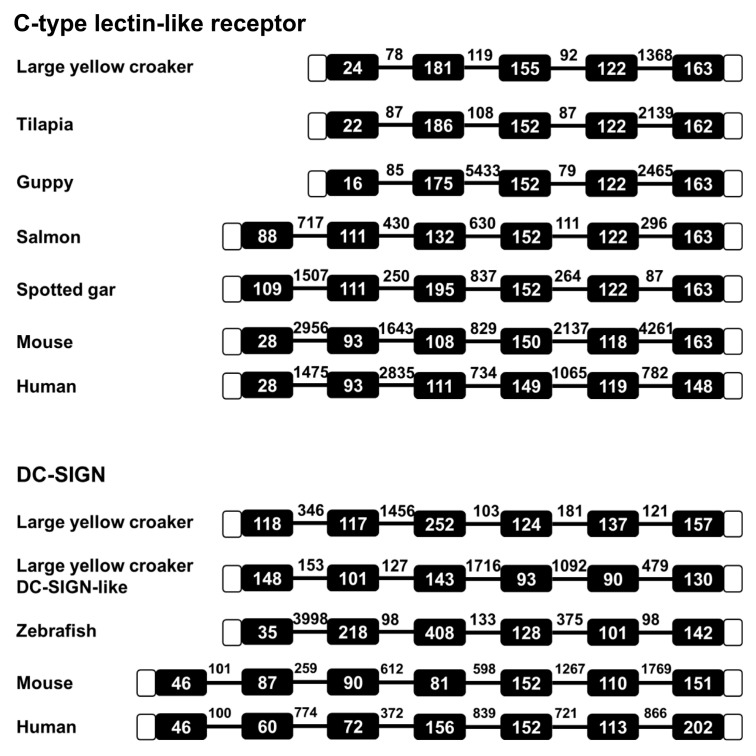
Genomic structure analyses of CTLR and DC-SIGN genes from fish and mammals. Exons are represented by closed boxes, and introns are represented by horizontal lines. Open boxes indicate UTR regions. The nucleotide length is shown in boxes and above lines. The genomic DNA sequences (and their accession numbers) are taken from GenBank database: CTLR: large yellow croaker, KQ041363 (EH28_17225); tilapia, NC_022209; guppy, NC_024346; salmon A, NM_001123579; spotted gar, NC_023204; mouse, NC_000072.6; human, AC092746.9. DC-SIGN: large yellow croaker, KQ041008; DC-SIGN-like, KQ041102; zebrafish, NC_007121.6; human, NC_000019.10; mouse, NC_000074.6.

The genomic sequence of LycCTLR is 2326 nt long, which consists of five exons and four introns, showing the same genomic organization as tilapia and guppy CTLRs. However, the salmon, spotted gar (*Lepisosteus oculatus*), mouse (*Mus musculus*), and human (*Homo sapiens*) CTLRs exhibited a different genomic structure, with six exons interrupted by five introns (Figure 3). In comparison, the genomic organisation of LycCTLR is divergent with that of large yellow croaker DC-SIGN gene which contains six exons and five introns. The same circumstances were also found in human and mouse, where both human and mouse DC-SIGN genes contain seven exons and six introns (Figure 3).

**Table 1:** Protein homology between LycCTLR and other C-type lectin domain-containing molecules.

Species	Common Name	Molecules	Identities (%)	NCBI Accession Number
		C-Type Lectin Receptor		
*Miichthys miiuy*	Miiuy croaker	C-type lectin-like receptor	88.4	AJF83862
*Oplegnathus fasciatus*	Barred knifejaw	C-type lectin receptor	62.3	ACY66647
*Lutjanus sanguineus*	Humphead snapper	C-type lectin receptor	61.9	AGT37609
*Epinephelus coioides*	Orange-spotted grouper	C-type lectin receptor	59.5	ACO06100
*Epinephelus akaara*	Hong Kong grouper	C-type lectin receptor	58.1	ACJ12598
*Oreochromis niloticus*	Tilapia	C-type lectin receptor	56.6	XP_003450637
*Salmo salar*	Salmon A	C-type lectin receptor A	45.6	NP_001117051
*Oncorhynchus mykiss*	Trout B	C-type lectin receptor B	42.8	NP_001153967
*Plecoglossus altivelis*	Ayu	C-type lectin receptor	35	CAZ39359
**Species**	**Common Name**	**Molecules**	**Identities (%)**	**NCBI Accession Number**
		**DC-SIGN**		
*Oryzias latipes*	Medaka	DC-SIGN	20.0	ADB55614
*Tetraodon nigroviridis*	Tetraodon	DC-SIGN	19.5	ADB55615
*Oreochromis niloticus*	Tilapia	DC-SIGN	19.1	XP_005463318
*Larimichthys crocea*	Large yellow croaker	DC-SIGN	18.7	KKF30589
*Danio rerio*	Zebrafish	DC-SIGN	14.0	ADB55613
**Species**	**Common Name**	**Molecules**	**Identities (%)**	**NCBI Accession Number**
		**l** **-SIGN**		
*Takifugu rubripes*	Fugu	l-SIGN	18.1	XP_003962680
*Cynoglossus semilaevis*	Tongue sole	l-SIGN	15.9	XP_008335870
*Miichthys miiuy*	Miiuy croaker	l-SIGN	15.4	AJF83861
*Oreochromis niloticus*	Tilapia	l-SIGN	14.4	XP_005456140
*Larimichthys crocea*	Large yellow croaker	l-SIGN	14.4	XP_010732178

### 2.2. Tissue Expression Analysis of LycCTLR Gene

**Figure 4:**
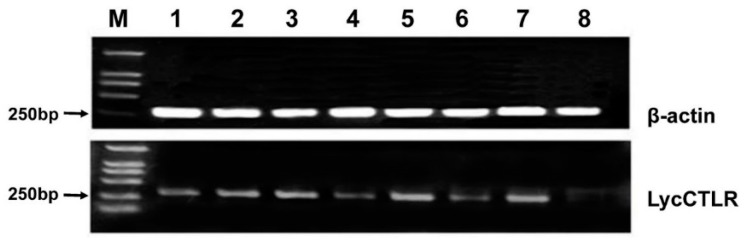
Expression analysis of LycCTLR gene in various tissues. Total RNA was extracted from eight tissues each from four normal fish, respectively, and RT-PCR was used to detect the expression levels of LycCTLR in various tissues. As a positive control for RT-PCR, β-actin was amplified to determine the concentration of templates. M: DNA Marker; 1. Gills; 2. Intestine; 3. Liver; 4. Kidney; 5. Heart; 6. Spleen; 7. Muscle; 8. Blood.

**Figure 5:**
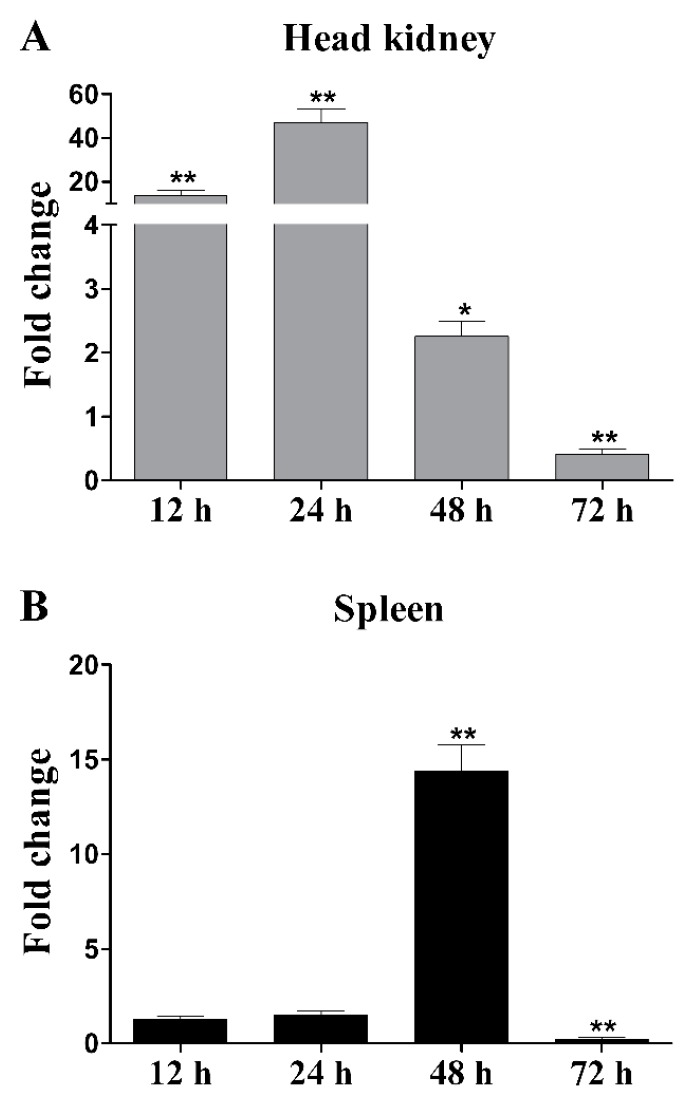
Expression analysis of LycCTLR gene in head kidney (**A**) and spleen (**B**) after inactivated trivalent bacterial vaccine induction. Head kidney and spleen were collected at 0, 12, 24, 48, and 72 h after bacterial vaccine induction, and total RNA was extracted for real-time PCR analysis. The relative expression level of LycCTLR was normalized by that of β-actin. The fold change was calculated as the average expression level of LycCTLR in the bacterial vaccine-challenged samples divided by that in the samples from phosphate buffered saline (PBS)-injected fish at each time point. Each experiment was performed in triplicate; error bars represent the standard error of the mean (SEM). The statistical significance of differences in gene expression was generated by two-tailed Student’s *t*-test compared with the data from PBS-injected group (* *p* < 0.05; ** *p* < 0.01).

LycCTLR mRNA was ubiquitously expressed in all examined tissues, with the highest levels in liver and heart, while the lowest level in blood (Figure 4). The LycCTLR gene was also expressed at a relatively low levels in head kidney and spleen. After induction with bacterial vaccine, the mRNA levels of LycCTLR in head kidney and spleen were significantly upregulated, and reached the peak at 24 and 48 h in head kidney and spleen, with 49.7- and 14.4-fold mRNA increases, respectively (Figure 5).

### 2.3. HemagglutinationActivity of LycCTLR

To characterize the biological activity of LycCTLR, LycCTLR gene was overexpressed in *E. coli* BL21 and rLycCTLR protein was purified by immobilized Ni-NTA rilotriacetic acid affinity chromatography (Figure S2). Then the purified rLycCTLR protein was used for hemagglutination assays. The results showed that rLycCTLR with 10 mM CaCl_2_ could induce the hemagglutination of mouse erythrocytes, rat (*Rattus norvegicus*) erythrocytes, and rabbit (*Oryctolagus cuniculus*) erythrocytes, but not induce the hemagglutination of tilapia erythrocytes. No agglutination was observed in the rLycCTLR without CaCl_2_ group, indicating that the hemagglutination of rLycCTLR was Ca^2+^-dependent. The agglutinating activity of rLycCTLR for rabbit erythrocytes and mouse erythrocytes was higher than that for rat erythrocytes with a minimal agglutination concentration of 4 μg/mL, compared with the minimal agglutination concentration of 64 μg/mL for rat erythrocytes (Table 2). However, the rLycactin had no agglutinating activity for these four animal erythrocytes under the same conditions.

**Table 2:** Hemagglutinating activity of rLycCTLR on animal erythrocytes.

Erythrocytes	Minimum Agglutinating Concentration of rLycCLTR (μg/mL)
Rabbit	4
Rat	8
Mouse	64
Tilapia	NA ^a^

^a^ NA: Nonagglutination at 64 μg/mL.

### 2.4. Sugar Binding SpecificityAssays

The sugar binding activity of rLycCTLR was examined by the inhibitory agglutination experiment using rabbit erythrocytes. The results showed that hemagglutinating activity of rLycCTLR was inhibited by the 10 types of sugars, except for d-mannose and d-arabinose, in a Ca^2+^-dependent manner (Table 3). d-galactose inhibited the hemagglutinating activity of rLycCTLR most effectively with a minimal concentration of 12.5 mM, followed by d-xylose, l-fucose, and cellobiose at 50 mM. The results suggested that rLycCTLR had a preference for galactose over other carbohydrates tested.

**Table 3:** Effects of saccharides on hemagglutinating activity of rLycCTLR.

Saccharides	Minimal Inhibitory Concentration (mM)
d-galactose	12.5
d-xylose	50
l-fucose	50
Cellobiose	50
Maltotriose	100
d-fructose	200
d-glucose	200
d-mannitol	200
Maltose	200
Sobitol	200
d-(−)-arabinose	NI ^a^
d-mannose	NI

^a^ NI: Not inhibited at 200 mM.

### 2.5. Bacterial Agglutinationassays

**Figure 6:**
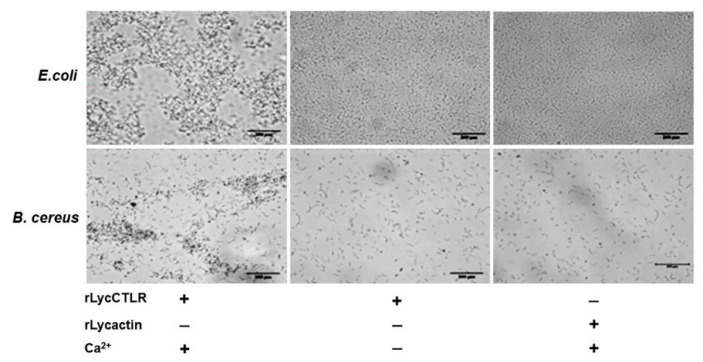
Bacterial agglutination caused by recombinant LycCTLR protein. For bacterial agglutination assays, eight strains of bacteria mentioned in the Materials and methods were tested. The results showed that the rLycCTLR at 64 μg/mL could agglutinate Gram-negative bacterium *E. coli* and Gram-positive bacterium *B. cereus*, but not the other six bacterial strains. No agglutinating activity was observed in the rLycCTLR without Ca^2+^ group and rLycactin control. The scale bar shown in the lower right is 200 μm.

The rLycCTLR at 64 μg/mL displayed the activity to agglutinate Gram-positive bacterium *B. cereus* and Gram-negative bacterium *E. coli* in the presence of Ca^2+^, but no agglutinating activity toward the other six bacterial strains, including *Pseudomonas fluoresces*, *Staphylococcus aureus*, *B.* *subtilis*, *Aeromonas* *hydrophila*, *Vibro alginolyticus*, and *Vibro parahaemolyticus*. Meanwhile, there was also no agglutinating activity observed in the rLycCTLR without Ca^2+^ group and rLycactin control, indicating that the agglutinating activity of rLycCTLR against *E. coli* and *B. cereus* was in a Ca^2+^-dependent fashion (Figure 6).

## 3. Discussion

In the present study, we have cloned and characterized a C-type lectin-like receptor (LycCTLR) from large yellow croaker. The deduced LycCTLR is a single transmembrane domain receptor with one typical CTLD at its C-terminus (Figure 1, residues 76–208). The CTLD contained four conserved cysteines (C3–C6 and C4–C5) and the WI/MGL motif, both important to maintain and stabilize the C-type lectin-like domain of C-type lectin, as well as two sugar binding motifs, EPD and WYD. The EPN in the CTLD is considered as a mannose-binding motif [20]. However, an EPD motif existed in the CTLD of LycCTLR instead of EPN, which might destroy the mannose-binding activity of LycCTLR. On the other hand, the Tyr^117^ in the WYD motif of the Atlantic salmon CTLRC, which has been identified to be the binding site of monosaccharides with preference for binding to d-mannose over for d-galactose [21], is also found in the corresponding WYD motif of LycCTLR (Try^195^, Figure 1). Additionally, LycCTLR also contained the residues essential for binding Ca^2+^ at sites 1 and 2 (Figure 1). These results indicated that LycCTLR is a novel Ca^2+^-dependent lectin-like receptor.

Studies on the tissue expression of fish CTLRs under normal or stimulated conditions have been reported [10,12,13,16,17]. In Atlantic salmon, three CTLR (CTLRA, B, and C) genes were expressed in most tissues of healthy fish, and their transcriptional levels were upregulated in the liver post-infection with *A. salmonicida* [10]. In grass carp (*Ctenopharyngodon idella*), mannose receptor C type 1 gene was obviously upregulated in the spleen, head kidney, liver, and intestine following *A. hydrophila* infection [13]. In zebrafish, mannose receptor mRNA was ubiquitously expressed in all examined tissues, and significantly increased in several examined tissues after *A. sobria* challenge [12]. In ayu, two CTLR molecules, PaCD209L and PaCTLRC, were reported to be significantly upregulated in each tested tissue upon *V. anguillarum* infection [16,17]. In this study, LycCTLR mRNA was also expressed in all examined tissues (Figure 4), and its expression levels were significantly increased in head kidney and spleen following inactivated bacterial vaccine stimulation (Figure 5). These results suggested that fish CTLRs may play an important role in antibacterial immune response.

To characterize hemagglutination activity of LycCTLR, the purified rLycCTLR protein was used for hemagglutination assays. The results showed that rLycCTLR could effectively agglutinate rabbit, mouse, and rat erythrocytes in the presence of Ca^2+^. This agglutination of animal erythrocytes by rLycCTLR was species-specific, since rLycCTLR showed no agglutination activity for tilapia erythrocytes. A similar situation was also found in the studies on a shrimp (*Penaeus monodon*) C-type lectin PmLec, where PmLec agglutinated rabbit, cattle, rat, and pig erythrocytes, but not human and mouse erythrocytes [22]. The inhibitory hemagglutination assays further demonstrated that among the carbohydrates tested, rLycCTLR had a preference for galactose over other carbohydrates (minimal inhibiting concentration of 12.5 mM), whereas rLycCTLR showed no affinity with mannose, which was inconsistent with the findings in ayu PaCTLRC and Atlantic salmon CTLRC [17,21]. The divergence in carbohydrate-binding activity of these fish lectins suggested that they may have different roles [23].

C-type lectins have essential roles in non-self-recognition and clearance of pathogenic microbes by binding to microbial surface carbohydrates [24]. In ayu, PaCTLRC showed agglutinating activity to all tested bacteria, including four Gram-negative bacteria (*V. parahaemolyticus*, *V. anguillarum*, *E. coli*, and *A. hydrophila*) and three Gram-positive bacteria (*Listeria monocytogenes*, *S. iniae* and *S. aureus*), in presence of Ca^2+^ [17]. In this study, rLycCTLR only displayed the agglutinating activity against Gram-positive bacterium *B. cereus* and Gram-negative bacterium *E. coli* in a Ca^2+^-dependent manner (Figure 6). Interestingly, *E. coli* and *B. cereus* both are main food-borne pathogenic bacteria that inhabit and contaminate aquaculture water and feed [25,26]. These results suggested that LycCTLR might be involved in eliminating these pathogens from contaminated water environment and feed. Of course, further research is required to elucidate the mechanism of LycCTLR in clearance of these pathogenic bacteria.

Conclusion: a novel C-type lectin-like receptor (LycCTLR) was cloned from large yellow croaker. LycCTLR was constitutively expressed in all tested tissues and its expression levels could be significantly upregulated following bacterial vaccine stimulation in the head kidney and spleen. Recombinant LycCTLR protein displayed not only the hemagglutinating activity and a preference for galactose, but also the agglutinating activity against two food-borne pathogens *E. coli* and *B. cereus* in a Ca^2+^-dependent manner. On the whole, our results reveal that LycCTLR is a potential galactose-binding C-type lectin and could serve as a pattern recognition receptor (PRR) involved in the antibacterial activity in fish.

## 4. Materials and Methods

### 4.1. Fish and Induction Experiment

Large yellow croakers (weight: 200 ± 13.5 g) were obtained from marine culture farm at Lianjiang, Fujian, China. Twenty normal fish in an aerated seawater tank (25 °C) were injected intramuscularly with inactivated trivalent vaccine at a dose of 0.5 mL/200 g fish after three days of acclimatizing. The inactivated trivalent vaccine containing 1.0 × 10^8^ colony-forming units·mL^−1^ of *A.* *hydrophila*, *V.* *alginolyticus*, and *V.* *parahaemolyticus* each, was prepared as previously described [27]. The control group was injected with same dose of sterilized PBS (pH 7.4). Head kidney and spleen tissues were collected from four fish at 0, 12, 24, 48, and 72 h after induction, and frozen in liquid nitrogen immediately.

### 4.2. Cloning of the LycCTLR

The 3′ and 5′ RACE-PCR were performed to obtain the full length cDNA of LycCTLR, using 3′ and 5′-RACE Kit (TaKaRa, Dalian, China). Primers (Table S1) for the RACE-PCR were designed from LycCTLR expressed sequence tag (EST, CX348992) [28]. All the resulting PCR product was sequenced and used to assemble the complete cDNA of LycCTLR. To obtain the genomic DNA sequence of LycCTLR, primers (CTLR-gF and CTLR-gR) were designed from predicted LycCTLR genomic sequence (EH28_17225) from large yellow croaker genome data [29]. Thirty nanograms of large yellow croaker muscle genomic DNA were used in PCR as template. PCR product was cloned and sequenced.

### 4.3. Sequence Analysis and Database

Multiple sequence alignment was performed by Clustal X2 programand colored using BoxShade [30]. The sequence identities were calculated using Clustal Omega [31]. Protein structures of CTLR genes were predicted using SMART [32]. Transmembrane helices was predicted by TMHMM Server v.2.1 [33]). MEGA version 6.0 was used to construct the phylogenetic tree with a neighbor-joining method [19]. Spidey (mRNA to genomic alignment) was used to analyze genomic structures of *CTLR* genes.

### 4.4. Tissue Expression Analysis of LycCTLR Gene

Total RNA was extracted from gills, muscle, intestine, liver, heart, kidney, spleen, and blood from four normal fish using Trizol Reagent (Invitrogen, Carlsbad, CA, USA). Two micrograms of each total RNA was used to synthesize first strand cDNA, respectively. RT-PCR was processed with primers of CTLR-DistF and CTLR-DistR (Table S1). PCR conditions consisted of 3 min at 94 °C, followed by 30 cycles of 30 s at 94 °C, 30 s at 56 °C, and 45 s at 72 °C, and a final incubation at 72 °C for 5 min. The β*-actin* (Table S1) was amplified to determine the concentration of each template, whose expression level is not influenced by bacterial vaccine [34].

### 4.5. Expression Modulation Analysis of LycCTLR Gene

Total RNA was extracted from head kidney and spleen tissues of four fish sampled at 0, 12, 24, 48, and 72 h after induction above. About 1 μg of each RNA was reverse-transcribed to the first strand cDNA. The β*-actin* was amplified with the actin-F and actin-R, as an internal control. Real-time PCR was performed using SYBR^®^ *Premix ExTaq™* (TaKaRa, Dalian, China) on Rotor-Gene 6000 (Corbett, Sydney, Australia) with primer set of CTLR-DistF and CTLR-DistR (Table S1). Cycling conditions were 95 °C for 3 min, followed by 40 cycles of 95 °C for 20 s, 56 °C for 20 s, and 72 °C for 20 s. The expression level of LycCTLR gene was normalized by that of β*-actin* using the 2^−ΔΔ*C*t^ method [35]. The fold change was calculated as the average expression level of LycCTLR in the bacterial vaccine-challenged samples divided by that in the samples from PBS-injected fish at each time point. Each experiment was repeated three times.

### 4.6. Expression and Purification of Recombinant LycCTLR

Recombinant LycCTLR (rLycCTLR) including the CTLD domain was expressed as described previously [36]. Briefly, the fragment of LycCTLR gene (residues 120–437) was amplified with primers of CTLR-RF and CTLR-RR (Table S1), and inserted into *Eco*R I/*Hin*d III-digested vector pET-His. The plasmid pET-LycCTLR was transformed into the competent cells of *E. coli* BL21 (DE3) (Invitrogen, Carlsbad, CA, USA). The rLycCTLR protein was expressed by 0.2 mM IPTG induction at 37 °C for 4 h. The rLycCTLR protein was purified by NI-NTA rilotriacetic acid affinity chromatography under denaturing conditions using the ProBond^TM^ Purification System (Invitrogen, Carlsbad, CA, USA). The proteins were refolded in 150 mM NaCl, 20 mM Tris-HCl, 0.5 mM reduced glutathione, and 0.1 mM oxidized glutathione overnight at 4 °C, then dialyzed against PBS, filtered with a sterile 0.2 μm filter, and stored at −80 °C. Protein concentration was quantified using Bradford assay by Nanodrop 1000 (Thermo Fisher Scientific, Wilmington, DE, USA). Recombinant large yellow croaker β-actin (rLycactin) was expressed and purified in the same system as a control.

### 4.7. Hemagglutination Assays

Hemagglutination assays were carried out according to the method described previously [22]. Briefly, two-fold serial dilutions of rLycCTLR protein solution in Tris-buffered saline (TBS) (100 mM NaCl, 20 mM Tris-HCl, pH 7.5) or TBS containing 10 mM CaCl_2_ (TBS-Ca) were prepared with a concentration range from 128 to 1 μg/mL. Twenty-five microliters of each diluted solution was then mixed with an equal volume of 2% erythrocyte suspensions from rabbit, rat, mouse (obtained from Xiamen University Laboratory Animal Central, Xiamen, Fujian, China), and tilapia (prepared by our laboratory) in a 96-well microtiter plate. After 1 h of incubation at 25 °C, erythrocyte hemagglutination was observed. Hemagglutinating activity to different animal erythrocytes of rLycCTLR was expressed as the minimum protein concentration that is required to agglutinate 2% animal erythrocytes. Hemagglutinating assays using rLycactin protein were also performed as negative controls. Each experiment was repeated three times.

### 4.8. Sugar Binding Assays

Serial dilutions (25 μL) of d-galactose, d-glucose, d-mannose, maltose, d-xylose, l-fucose, cellobiose, sobitol, d-fructose, d-mannitol, d-arabinose, and maltotriose (Sigma, Saint Louis, MO, USA) each in TBS-Ca, were mixed with 25 μL of rLycCTLR (8 μg/mL) and incubated at 37 °C for 30 min. Then, 2% suspension of rabbit erythrocytes was added and incubated at 25 °C for 30 min. Finally, the inhibition of agglutination was observed. Inhibitory effect was exhibited as the minimal concentration of each carbohydrate required for complete inhibition of hemagglutination activity of rLycCTLR. The assays were repeated three times.

### 4.9. Bacterial Agglutination Assays

For bacterial agglutination assays, four Gram-positive bacteria (*P.* *fluoresces*, *B. cereus*, *S.* *aureus*, and *B.* *subtilis*) and four Gram-negative bacteria (*V. parahaemolyticus*, *V. alginolyticus*, *A. hydrophila*, and *E. coli*; all these eight bacteria were kindly gifts from Xuanxian Peng, Sun Yat-sen University, Guangzhou, Guangdong, China) were cultured in Luria-Bertani (LB) medium overnight. Cells were harvested and resuspended to 2.5 × 10^9^ cells/mL in TBS. The microorganism/TBS solution (25 μL) was incubated with 25 μL rLycCTLR/TBS (300 μg/mL) at 25 °C for 1 h, containing 10 mM CaCl_2_. Agglutination of bacteria was examined by microscopy. Bacterial agglutination activity of rLycactin protein was analyzed as a negative control. All the assays were repeated three times.

## Supplementary Materials

Supplementary materials can be found at http://www.mdpi.com/1422-0067/16/12/26175/s1.
