# Supplementary material for: Molecular Characterization and Biological Effects of a C-Type Lectin-Like Receptor in Large Yellow Croaker (Larimichthys crocea)
Source: Int J Mol Sci. 2015 Dec 10;16(12):29631–42. doi: 10.3390/ijms161226175 (PMC4691118; doi:10.3390/ijms161226175)
Supplement: Supplementary file 1 [file ijms-16-26175-s001.pdf]

Supplementary Materials: Molecular Characterization and Biological Effects of a C-Type Lectin-Like Receptor in Large Yellow Croaker (*Larimichthys crocea*)

Jingqun Ao, Yang Ding, Yuanyuan Chen, Yinnan Mu and Xinhua Chen*

```
                                             GAAGTTGAAGTCTCTGCAAACTCACAAAAAA
1     ATGATGAACCTCATCAAAAAGAAAGGTGCCCTGGGAATCAGAGGACACGCAGTCCTCTTT
1      M  M  N  L  I  K  K  K  G  A  L  G  I  R  G  H  A  V  L  F
61    GTTTTCATCAGCCTTATGGTTTCTGTAGCTGCAGACAGTGCAGAGGGACAGGAGTCCTAT
21     V  F  I  S  L  M  V  S  V  A  A  D  S  A  E  G  Q  E  S  Y
121   CTAAAGCTGAGACTAGACCTTTTGAAGAAAAGCTACAGTAAGCTGTGCAGTGAATACACC
41     L  K  L  R  L  D  L  L  K  K  S  Y  S  K  L  C  S  E  Y  T
181   AACCTGGCACAGAACTGCTCAGTTCCAGTGCCTAACTGTTACGAGTGTCCTGACGACAAA
61     N  L  A  Q  N  C  S  V  P  V  P  N  C  Y  E  C  P  D  D  K
241   TGGCTTCTAGTAGGGGACCAATGCCTCCTCCTCGAGACTGACAGGAACGACTGGCTTAAT
81     W  L  L  V  G  D  Q  C  L  L  L  E  T  D  R  N  D  W  L  N
301   AGTTCAAAAAAGTGTGAAGAGATGGGCGCCCATCTTGCCATCTTGACCACCACAGAACAG
101    S  S  K  K  C  E  E  M  G  A  H  L  A  I  L  T  T  T  E  Q
361   CATGAAGCTGTGGAGAAAGAAGGCAGAATGCTCGGTGGGTTATACACATTCTACTGGATG
121    H  E  A  V  E  K  E  G  R  M  L  G  G  L  Y  T  F  Y  W  M
421   GGACTGACTGACATTGAGAAAGAAGGAGAGTGGAAATGGGTGGACAACTCAATAGTTAAA
141    G  L  T  D  I  E  K  E  G  E  W  K  W  V  D  N  S  I  V  K
                ◆     ◆
481   AACACACACTGGAAGGTTGGGACATCAGAACCAGACAACAACCAGTCTGGTGGGGAAGAG
161    N  T  H  W  K  V  G  T  S  E  P  D  N  N  Q  S  G  G  E  E
                                  ◇     ◇     ◆
541   GGAGAGGACTGTGCGGTAGTGGACAGCTACACTCAGAGCTGGTACGATGTTCCCTGCTCC
181    G  E  D  C  A  V  V  D  S  Y  T  Q  S  W  Y  D  V  P  C  S
          ◇  ◆                                   ◇  ◇
601   TACTTGTATCCACGAATCTGTCAGGTCAACGCCAAACTGCTCAAGTGAGCCTCTCCGCAC
201    Y  L  Y  P  R  I  C  Q  V  N  A  K  L  L  K  *
661   CACTGCAGGACCACCACTCAATAACAATAATTGATAGCTATCGATTCTTTATTGAAAAAA
721   CTTCAGGCATCAATCTACAAATAAACAATTCTGACTCTAAAAAAAAAAA
```

Figure S1. Nucleotide and deduced amino acid sequences of LycCTLR cDNA. In the nucleotide and deduced amino acid sequences of LycCTLR cDNA, a typical mRNA polyadenylation signal (AATAAA) are shown in italic in the 3′-UTR. The transmembrane domain is boxed and the intracellular domain is marked by broken line. The C-type lectin-like domain predicted by SMART is underlined. The conserved cysteine residues are highlighted in dark grey. Ca^{2+} binding site 1 are marked with solid diamond. Ca^{2+} binding site 2 are marked with hollow diamond.

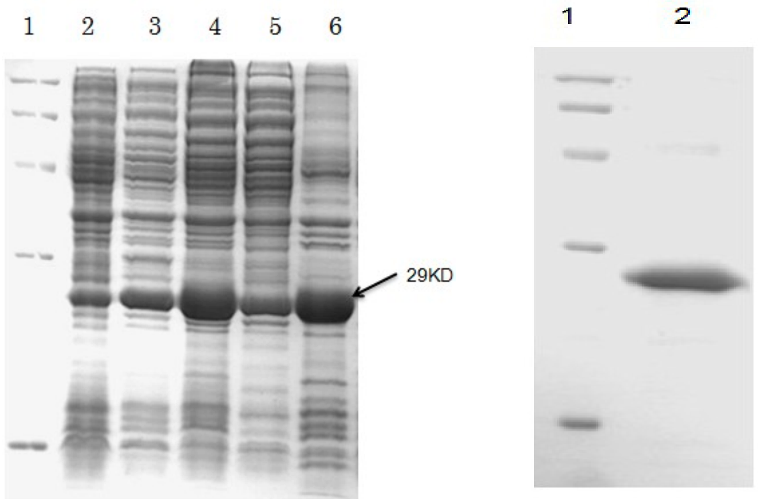

(**a**) (**b**)

Figure S2. Production and purification of recombinant LycCTLR protein. (**a**) Lane 1: protein molecular weight marker; Lane 2: induced pET-His in *E. coli* BL21; Lane 3: non-induced pET-LycCTLR in *E. coli* BL21; Lane 4: induced pET-LycCTLR in *E. coli* BL21; Lane 5: supernatant from induced pET-LycCTLR in *E. coli* BL21; Lane 6: inclusion body from induced pET-LycCTLR in *E. coli* BL21; (**b**) Lane 1: protein molecular weight marker; Lane 2: purified rLycCTLR protein.

Table S1. Oligonucleotide primers used for cloning and expression analyses.

Primer Name	Nucleotide Sequence (5′→3′)	Purpose
5′ outer	ACACTCGTAACAGTTAGGCACT	RACE PCR
5′ inner	AGAAACCATAAGGCTGATGAAA	
3′ outer	GAAATGGGTGGACAACTCAATA	
3′ inner	GGGACATCAGAACCAGACAACA	
CTLR-gF	GAAGTCTCTGCAAACTCACAAA	Genome cloning
CTLR-gR	GTCAGAATTGTTTATTTGTAGAT	
CTLR-DistF	CGGAATTCGAGGGACAGGAGTCCTATCTA	Real-time PCR
CTLR-DistR	CCCGGACTTTCACTTGAGCAGTTTGGCGT	
CTLR-RF	CCGGAATTCATGATGAACCTCATCAAAAA	Recombinant expression
CTLR-RR	CCCAAGCTTTCACTTGAGCAGTTTGGCGT	
Actin-F	GACCTGACAGACTACCTCATG	β-actin amplification
Actin-R	AGTTGAAGGTGGTCTCGTGGA	
